# Clinical Microbial Identification of Severe Oral Infections by MALDI-TOF Mass Spectrometry in Stockholm County: an 11-Year (2010 to 2020) Epidemiological Investigation

**DOI:** 10.1128/spectrum.02487-22

**Published:** 2022-11-24

**Authors:** Khaled Al-Manei, Mahin Ghorbani, Sabrina Naud, Kholod Khalil Al-Manei, Michał J. Sobkowiak, Bodil Lund, Gulsen Hazirolan, Margaret Sällberg Chen, Volkan Özenci

**Affiliations:** a Unit of Oral Diagnostics and Surgery, Department of Dental Medicine, Karolinska Institute t, Huddinge, Sweden; b Division of Endodontics, Department of Restorative Dental Science, College of Dentistry, King Saud University, Riyadh, Saudi Arabia; c Medical Unit of Plastic Surgery and Oral and Maxillofacial Surgery, Department of Oral and Maxillofacial Surgery and Jaw Orthopedics, Karolinska University Hospital, Stockholm, Sweden; d Division of Clinical Microbiology, Department of Laboratory Medicine, Karolinska Institute t, Huddinge, Sweden; e Department of Clinical Microbiology F 72, Karolinska University Hospital, Huddinge, Sweden; Tainan Hospital, Department of Health, Executive Yuan

**Keywords:** oral microbiota, microbiology trend, dental abscess, osteomyelitis, periodontitis, matrix-assisted laser desorption-ionization, dental infection

## Abstract

Growing evidence suggests that oral infections can modify the course of systemic diseases. To date, epidemiological data on microbial oral infections are scarce. Here, we performed a comprehensive analysis of the trend and microbial diversity in oral infection specimens referred for clinical microbiology analysis from 2010 to 2020. The microbes were isolated by culture and were identified via matrix-assisted laser desorption ionization-time of flight mass spectrometry technology (MALDI-TOF MS) throughout the study period. A total of 1,014 referred samples from dental clinics in Stockholm County with dentoalveolar abscesses and jaw osteomyelitis being the main reason were identified. Overall, the microbial composition was dominated by *Firmicutes* (51%), followed by *Bacteroidetes* (19%), *Proteobacteria* (12%), and *Actinobacteria* (5%). At the genus level, Streptococcus spp. (36%), *Prevotella* spp. (18%), and Staphylococcus spp. (11%) were among the most frequently reported. Interestingly, a strong increase in trend was noted for Streptococcus anginosus, Streptococcus mitis, Streptococcus sanguinis, Eikenella corrodens, *Actinomyces* spp., Aggregatibacter aphrophilus, Staphylococcus epidermidis, and Granulicatella adiacens during the study time (*R* = 0.66 to 0.89, *P* < 0.05), and a minor increase was noted for Enterococcus faecalis and Klebsiella spp., whereas steady levels were noted for most of the others. The present study shows the diversity of bacteria that have been involved in dental infections during the last decade in the capital of Sweden, as well as the emerging oral microbiota trend, with clear clinical implications on the oral-systemic link.

**IMPORTANCE** Oral diseases and associated microbes are a risk factor for systemic diseases and can change the courses of these diseases. To date, epidemiological data on microbial oral infections are scarce, and longitudinal reports are lacking. We present for the first time the microbial composition of severe oral bacterial infections determined via the MALDI-TOF mass spectrometry technique in a comprehensive study between 2010 and 2020 (11 years) in Stockholm County. The trend and microbial diversity of oral infections were analyzed on referred clinical microbiological samples and were processed by standardized protocols. Trend increase was noted for Streptococcus anginosus, Streptococcus mitis, Streptococcus sanguinis, Eikenella corrodens, *Actinomyces* spp., Aggregatibacter aphrophilus, Staphylococcus epidermidis, Granulicatella adiacens, Enterococcus faecalis, and Klebsiella spp. Our results provide new insights into the diversity and trend of oral microbiota that were involved in serious oral infections over the past decade in the capital of Sweden and may influence the oral-systemic link.

## INTRODUCTION

The oral cavity is a crucial component of the human body and is essential to overall health and well-being. Despite advancements in oral health care systems, oral diseases are still listed among the top leading noncommunicable diseases (1). Oral disease manifestations not only contribute to the increasing disease burden in our societies, as noted in the recent Global Burden of Diseases, Injuries, and Risk Factors Study (GBD) report, but also raise medical concerns (2). Especially in aging, socially deprived, or medically deprived communities, common oral/dental diseases, such as dental caries, periapical periodontitis, soft tissue abscesses, and periodontal diseases, may progress and exacerbate the burden of other diseases (2). Consequently, serious infections might be developed, and these might require emergency and urgent care.

Oral infections commonly originate from an odontogenic source and are typically caused by opportunistic microbes (3–5) that disseminate via deep dental caries, endodontic, periodontal diseases, or tissue infections (6). Deep dental caries and endodontic infections are the main causes of dental abscesses (6), whereas periodontal diseases, including periodontitis, or pericoronitis cause periodontal abscesses (7). Clinically, the effects range from localized abscesses to deep head and neck space infections, which may require hospitalization (8). Jaw osteomyelitis and odontogenic maxillary sinusitis constitute other serious complications following odontogenic infections (9, 10). Osteonecrosis is also frequently associated with immunosuppression (11) as well as head/neck radiation, and it can be medically induced (12–15).

Accumulating research suggests that oral diseases are an increasingly common risk factor for systemic diseases (16) and are an emerging challenge for health care (17, 18). Oral pathobionts or harmless commensals may “turn against” the host and cause tissue damage or reactivity if an opportunity is present (19). In this context, severe infections in the oral cavity and associated microbes are important to mine and monitor. Earlier studies using culture-based methods reported mixed oral microbiota, which mainly consisted of facultative anaerobes with annotations limited to the Streptococcus spp. and strict anaerobes, particularly *Prevotella* and *Fusobacterium* spp. (4).

Matrix-assisted laser desorption ionization-time of flight mass spectrometry (MALDI-TOF MS) became a standard clinical microbiology method nearly a decade ago and is the method of choice for the identification of microorganisms in clinical laboratories. MALDI-TOF MS is an accurate, fast, and cost-effective technology developed for routine clinical diagnostics (20). It is highly sensitive to a variety of clinical samples and allows microorganisms to be identified using their proteomic fingerprints, which are primarily made up of ribosomal proteins (20). To date, the usage of this technique to describe the microbiological profile of oral infections at a population level has not been reported.

Here, we conducted an 11-year retrospective epidemiological study in Stockholm County to determine the microbial profile longitudinally of severe oral infection samples that were referred to the Karolinska University Clinical Microbiology Laboratory.

## RESULTS

### Demographic distribution.

Samples were analyzed from a total of 1,014 patients. The average age was 56.34 (± a standard deviation of 21.70) years. 469 were female, and 545 were male. Dentoalveolar abscess was the most prevalent clinical diagnosis, followed by jaw osteomyelitis (Table 1). Adults in age groups ≥60, 40 to 59, or 18 to 39 each accounted for around 30% of the total patients, and 5.5% were from individuals who were <18 years old. A total of 102 bacteria species were reported during the entire study period, with a range of 1 to 7 species per sample. Proportions of mono-respective polymicrobial culture findings were similar across indicated subgroups, except for the jaw osteonecrosis subgroup, in which polymicrobial cultures were more likely to occur, as noted by a logistic regression analysis, (*P* < 0.001).

**TABLE 1:** Multivariable logistic regression analysis assessing clinical diagnosis, gender, age, and sample material to predict the occurrence of polymicrobial culture^*a*^

Variable	Sample (%)	Microbial culture (%)^*b*^		Coe.	SE	OR (%)	CI (95%)	*P* value
		Mono-	Poly-					
Clinical diagnosis								
Dentoalveolar abscesses	55.6	50.5	49.5	-	-	1	-	Reference
Jaw osteomyelitis	15.3	51.9	48.1	0.136	0.223	1.14	0.74 to 1.77	0.540
Jaw osteonecrosis	8	30.9	69.1	0.952	0.294	2.59	1.46 to 4.61	0.001*
Odontogenic maxillary sinusitis	4.1	56.1	43.9	0.245	0.431	1.28	0.55 to 2.98	0.570
Periradicular pathosis	3	36.7	63.3	0.873	0.480	2.39	0.94 to 6.13	0.068
Periodontal diseases	2	60	40	−0.322	0.512	0.73	0.27 to 1.98	0.530
Age in years								
≥60	33.5	50.3	49.7	-	-	1	-	Reference
40 to 59	32	52.7	47.3	0.100	0.170	1.11	0.79 to 1.54	0.557
18 to 39	29	45.8	54.2	0.131	0.167	1.14	0.82 to 1.58	0.435
<18	5.5	53.9	46.2	−0.120	0.323	0.89	0.47 to 0.67	0.712
Gender								
Male	53.8	49.6	50.4	-	-	1	-	Reference
Female	46.3	51.4	48.7	0.012	0.130	1.01	0.78 to 1.31	0.930
Sample material								
Abscess	72	48.5	51.5	-	-	1	-	Reference
Mucosa	10	62.1	37.9	−0.483	0.307	0.62	0.33 to 1.12	0.116
Tissue	7	51.4	48.7	−0.424	0.347	0.65	0.33 to 1.29	0.222

a Coe., coefficient; SE, standard error; OR, odds ratio; CI, confidence interval.

b The dashed line (−) indicates that no value has been delegated to Coe., SE, or CI in the reference variables. *, a *P*-value with statistical significance (*P* < 0.5).

### Microbial composition and annual trend from 2010 to 2020.

**FIG 1:**
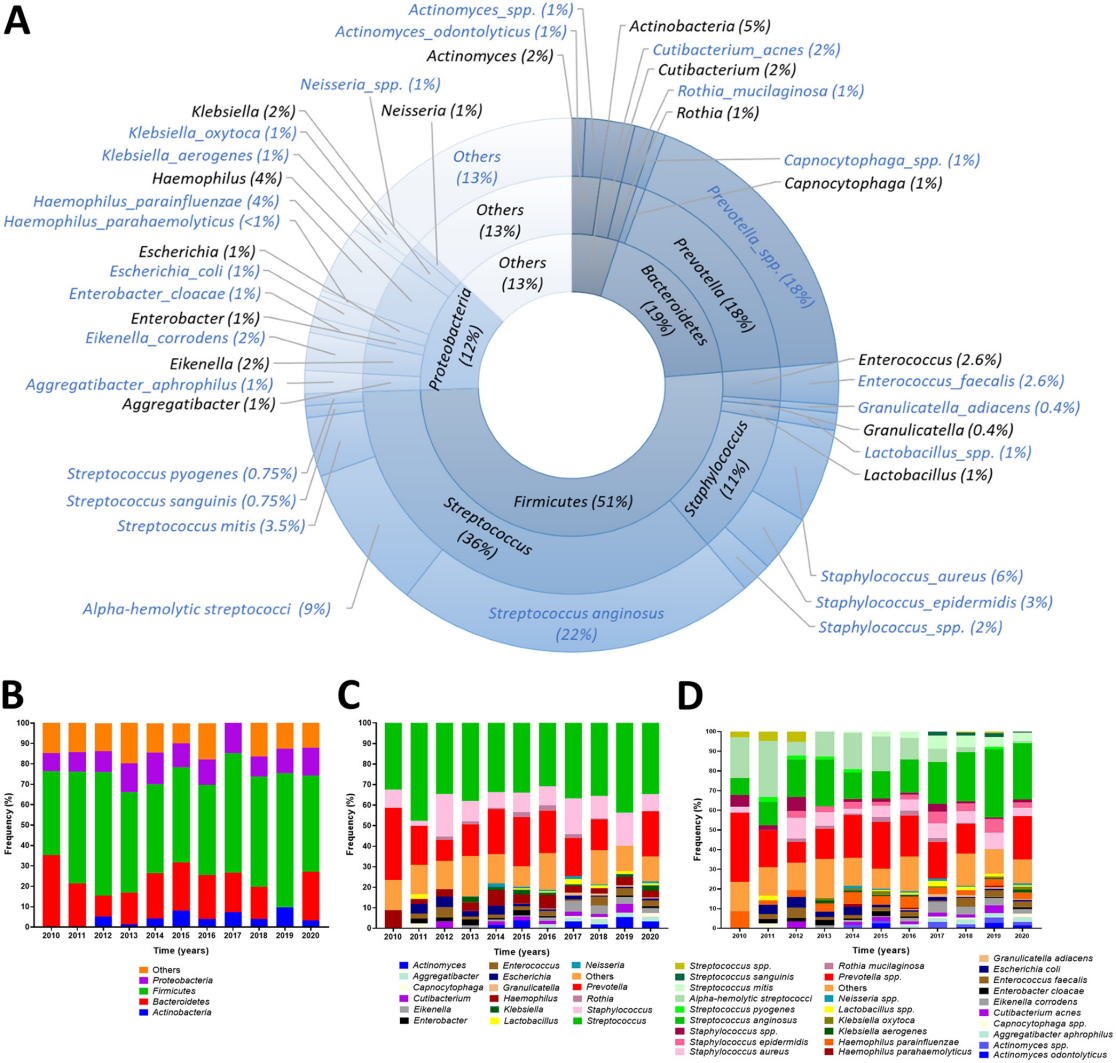
Overall microbes in oral infections. A global view of the oral bacteria reported during the entire 11-year study period, classified at the phylum, genus, and species levels, presented as a donut chart (A). Stacked bars illustrate the yearly frequency of the microbial phyla (B), genera (C), and species (D) reported for the oral infection samples. The data are expressed in percentages.

To investigate the microbial composition and trends of the identified bacteria, the data were arranged at the phylum, genus, and species levels. As shown in Fig. 1A, 4 phyla constituted the vast majority of the detected microbial species: in ranked order, *Firmicutes* (51%; genera Streptococcus, Staphylococcus, *Enterococcus*, *Lactobacillus*, and *Granulicatella*), *Bacteroidetes* (19%; genera *Prevotella*, and *Capnocytophaga*), *Proteobacteria* (12%; genera Haemophilus, *Eikenella*, Klebsiella, Escherichia, Enterobacter, *Aggregatibacter*, and *Neisseria*), and *Actinobacteria* (5%; genera *Actinomyces*, *Cutibacterium*, and *Rothia*); with the remaining 13% being unclassified. At the genus level, Streptococcus spp. and *Prevotella* spp. account for almost 50% of the reported bacteria, followed by Staphylococcus spp. (11%). The microbial distribution at the phylum, genus, and species levels also exhibited a predominance of Streptococcus spp., *Prevotella* spp., and Staphylococcus spp. across the 11 years included (Fig. 1B–D).

**FIG 2:**
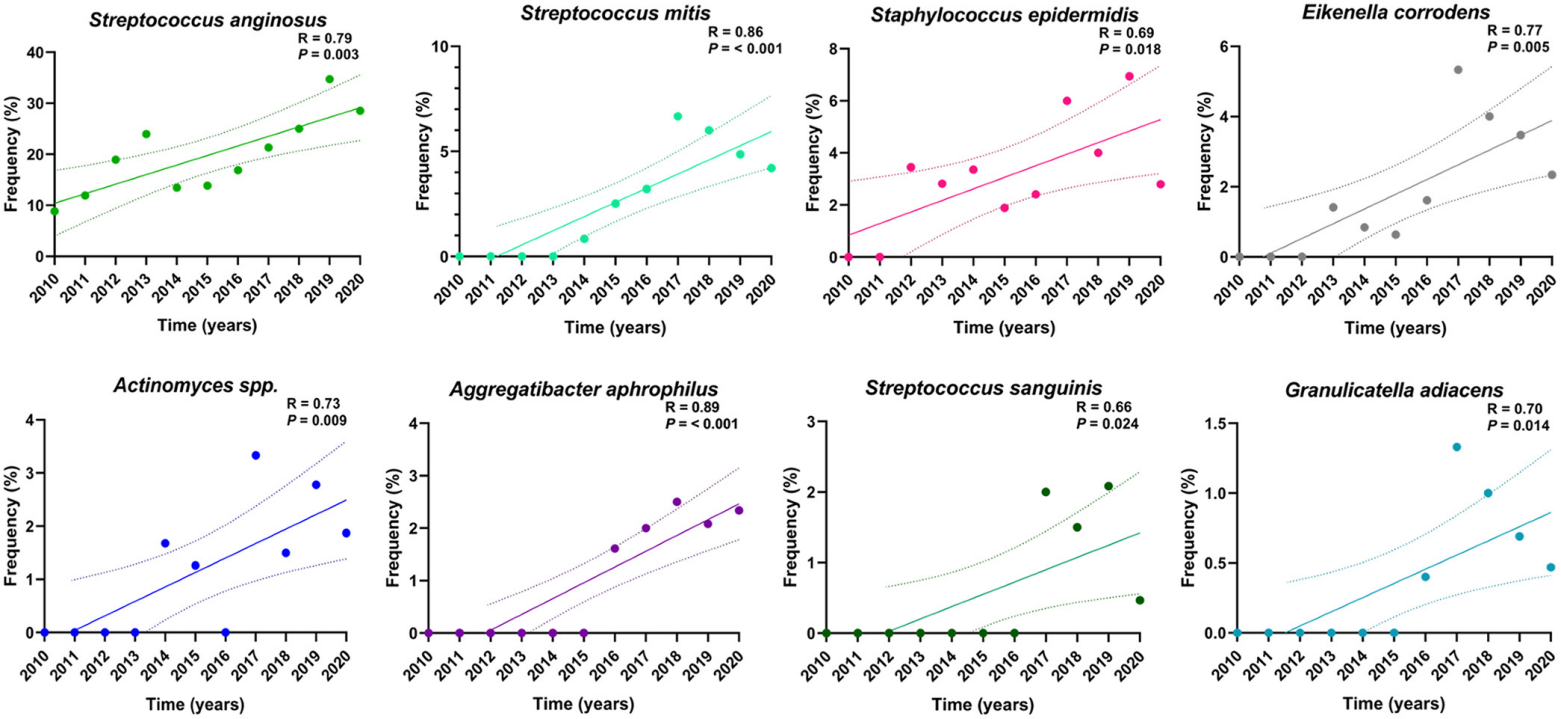
Trends of the reported microbes in oral infections over the 11-year study period. A linear regression analysis indicates a significant increase of Streptococcus anginosus, Streptococcus mitis, Streptococcus sanguinis, Eikenella corrodens, *Actinomyces* spp., Aggregatibacter aphrophilus, Staphylococcus epidermidis, and Granulicatella adiacens in oral infection samples. *P* values of <0.05 are considered to be indicative of a statistically significant result.

A subsequent trend analysis based on the annual data (Fig. 2) indicated that among the Streptococcus spp., the frequency of Streptococcus anginosus, Streptococcus mitis, and Streptococcus sanguinis had increased significantly (*R* = 0.79, *R* = 0.86, and *R* = 0.66, respectively; *P* < 0.05) (Fig. 2). Moreover, significant increases were also noted for Staphylococcus epidermidis (*R* = 0.69), Eikenella corrodens (*R* = 0.77), *Actinomyces* spp. (*R* = 0.73), Aggregatibacter aphrophilus (*R* = 0.89), and Granulicatella adiancens (*R* = 0.70; *P* < 0.05) (Fig. 2). A mild to moderate increase was also noted for Enterococcus faecalis, Klebsiella spp., *Capnocytophaga* spp., *Lactobacillus* spp., and *Neisseria* spp., (*R* = 0.30, *R* = 0.38, *R* = 0.16, *R* = 0.35, and *R* = 0.39, respectively; *P > *0.05) (Fig. S1). On the other hand, a moderate decline was found for *Prevotella* spp., *Haemophilus* spp., Escherichia coli, and Streptococcus pyogenes (*R* = −0.42, *R* = −0.70, *R* = −0.50, and *R* = −0.40, respectively; *P > *0.05) (Fig. S1).

### Microbial distribution and annual trend by Gram staining, morphology, and oxygen metabolism.

**FIG 3:**
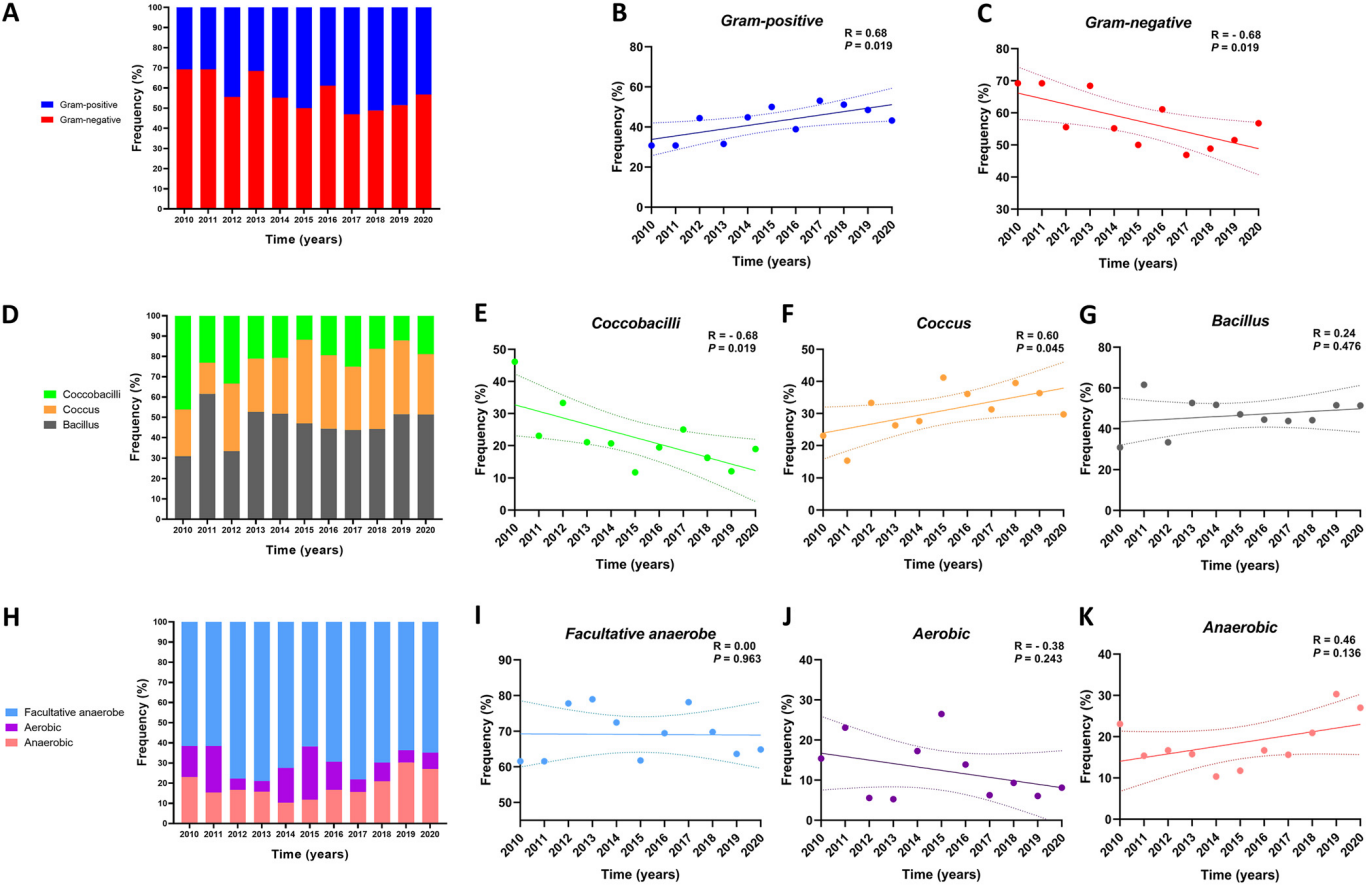
Distribution and trends of reported microbes of oral infections, as classified by Gram staining, morphology, and oxygen metabolism. Stacked bars illustrate the yearly frequencies of Gram-positive versus Gram-negative bacteria (A), coccobacilli, coccus, and bacillus (D), facultative anaerobe, aerobic, and anaerobic (H) bacteria that were detected in oral infection samples. A linear regression analysis indicates the trends of the Gram-positive (B), Gram-negative (C), coccobacilli (E), coccus (F), bacillus (G), facultative anaerobe (I), aerobic (J), and anaerobic (K) bacteria noted in oral infection samples over the 11-year study period. The data are expressed in percentages. *P* values of <0.05 are considered to be indicative of a statistically significant result.

Overall, the proportions of Gram-negative and Gram-positive bacteria were 55.4% and 44.6% over the past 11 years (Fig. 3A). The frequency of Gram-positive species increased significantly at the expense of Gram-negative species over the study period (*R* = 0.68; *P* < 0.05) (Fig. 3B and C). As for microbial morphology, bacilli accounted for 47% of the total reported microbes, followed by cocci (33%), and coccobacilli (20%) (Fig. 3D). While the frequencies of bacillus species remained stable over time (*R* = 0.24; *P* > 0.05), the frequency of the coccobacilli and cocci changed significantly over time (*R* = −0.68 and *R* = 0.60, respectively; *P* < 0.05) (Fig. 3E–G). As for oxygen metabolism, facultative anaerobes were the most frequently detected, followed by anaerobic and aerobic species (Fig. 3H). A trend analysis indicated no significant changes in either of these species (Fig. 3I–K).

### Microbial profiles by clinical diagnosis and sample material.

**FIG 4:**
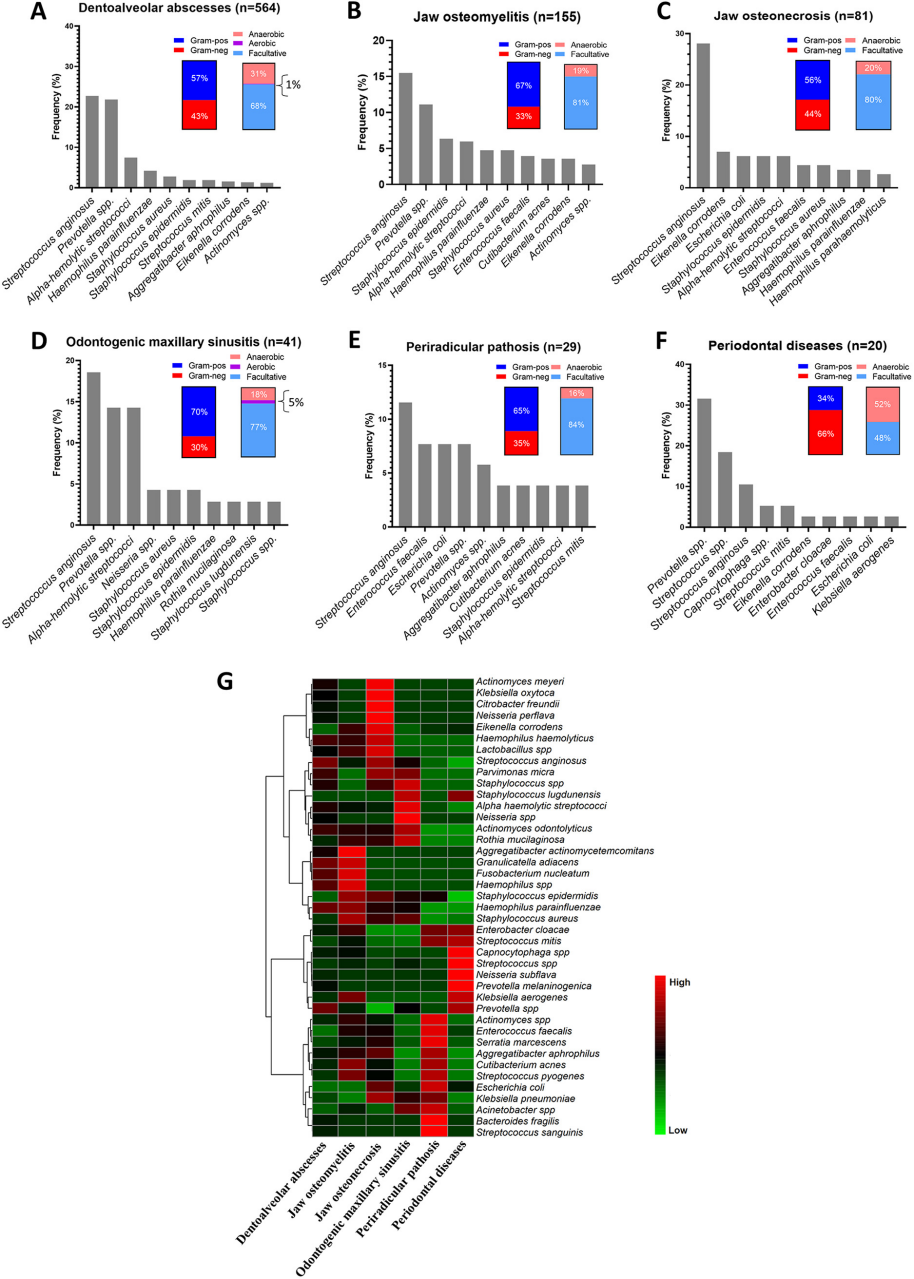
Microbial profile of oral infections based on their clinical diagnosis group. Bars illustrate the frequencies of the top-10 microbes reported in dentoalveolar abscesses (A), jaw osteomyelitis (B), jaw osteonecrosis (C), odontogenic maxillary sinusitis (D), periradicular pathosis (E), and periodontal diseases (F), respectively. Stacked bars in inserted panels indicate the proportions of Gram-positive versus negative, facultative anaerobe, aerobic, and anaerobic bacteria phenotypes, respectively. The data are expressed in percentages. (G) Hierarchical clustering heat map representation of the occurrence of bacterial species between different oral infections. Colors represent the level of species occurrence, which is indicated as low (light green) to high (light red), based on the Euclidean distance from the maximum level of each species among the diagnosed groups.

Among the 1,014 identified samples, 890 were assigned to six different clinical diagnoses: dentoalveolar abscesses, jaw osteomyelitis, jaw osteonecrosis, odontogenic maxillary sinusitis, periradicular pathosis, and periodontal diseases. 124 samples were listed as undiagnosed. The most frequently involved facial spaces were the submandibular site (43%), followed by the buccal site (38%), submental region (11%), palate (3%), canine (3%), and pterygomandibular region (2%). As shown in Fig. 4A–F and in Table 1, dentoalveolar abscesses accounted for 55.6% of the total samples, in which S. anginosus, *Prevotella* spp., and Haemophilus parainfluenzae were frequently noted. Interestingly, *S. anginosus* was also commonly associated with jaw osteomyelitis, jaw osteonecrosis, odontogenic maxillary sinusitis, and periradicular pathosis. Other frequent microorganisms in jaw osteomyelitis were S. epidermidis, H. parainfluenzae, Staphylococcus aureus, and E. faecalis. In periradicular pathosis samples, E. faecalis, E. coli, and *Prevotella* spp. were frequently detected, whereas in periodontal diseases, *Prevotella* spp. and Streptococcus spp. were detected in over 50% of the cases. Across the clinical diagnoses, five bacterial species were found to be in common: *S. anginosus*, S. aureus, E. faecalis, E. corrodens, and S. mitis.

Although a general predominance of Gram-positivity and facultative anaerobes was noted, the periodontal disease group frequently showed Gram-negativity as well as anaerobic species (Fig. 4F). A deeper examination via a heat map analysis on the microbial species between the disease conditions also showed a substantial interecology occurrence (Fig. 4G). Interestingly, while the strictly anaerobic Fusobacterium nucleatum and Aggregatibacter actinomycetemcomitans (a fastidious HACEK organism) were noted, they occurred mainly in cases of jaw osteomyelitis or dentoalveolar abscess. Other fastidious HACEK organisms, such as E. corrodens, H. haemolyticus, *H. parainfluenza*, and A. aphrophilus, also occurred mainly in jaw osteonecrosis and periradicular pathosis cases, and occasionally in cases of periodontal disease, as revealed by the clinical MALDI-TOF approach.

**FIG 5:**
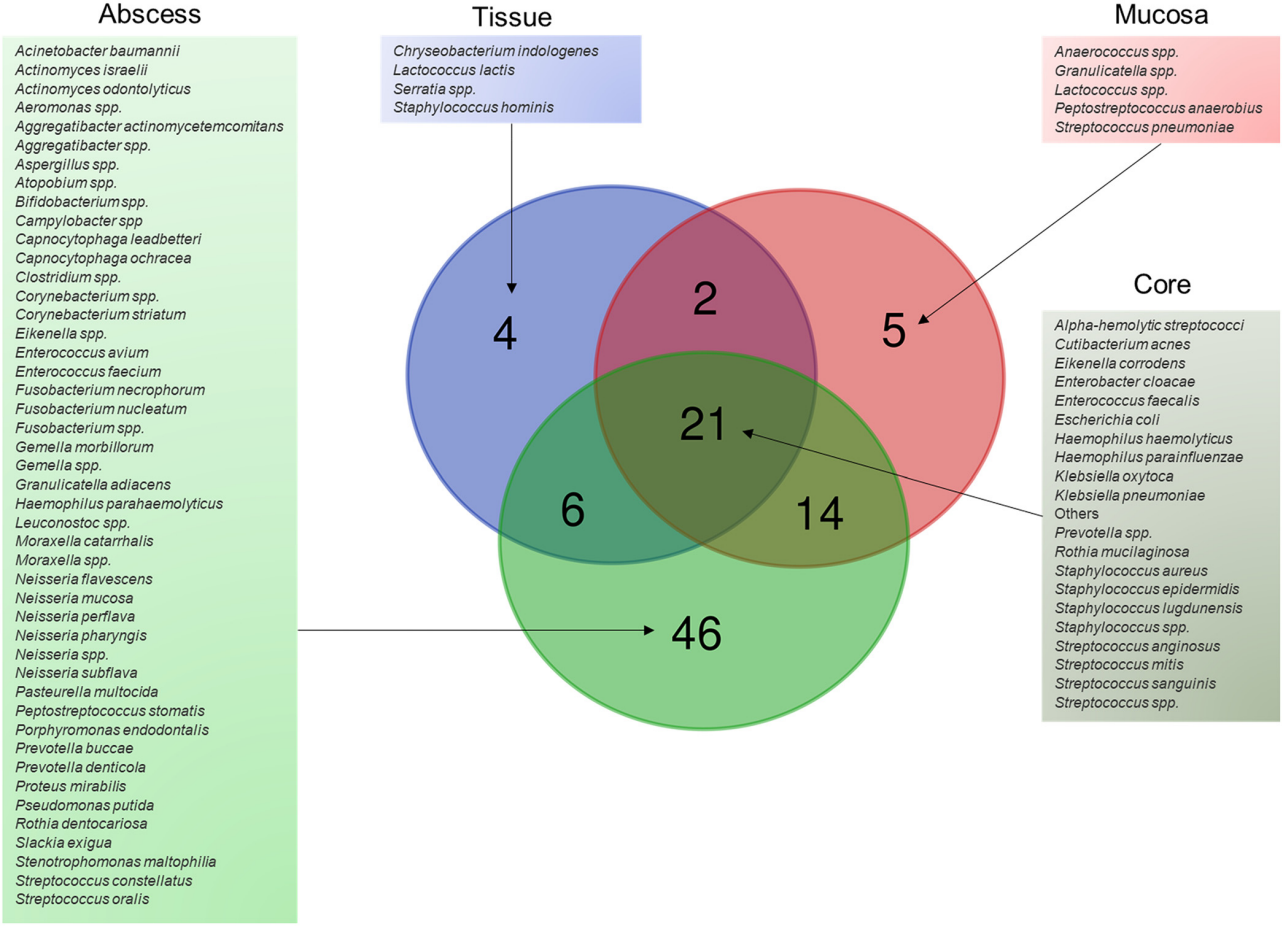
Unique and shared core microbiota in oral infections. The Venn diagram depicts the relation between the identified bacteria species, organized according to the indicated sampling materials. The overlapping subset indicates the shared core species found in all three sample types.

As for the sample material, abscesses were the most prevalent (72%), followed by mucosa swabs (10%), and tissue samples (7%). 11% of all samples were comprised of material of an unspecified origin. Interestingly, the Venn analysis presented in Fig. 5 revealed that 21 species are shared by all 3 sample types and could represent the core microbes of the oral infection samples reported in this study. Among the core members were E. corrodens, E. faecalis, *S. anginosus*, S. mitis, and S. epidermidis as well as members of gamma-proteobacteria, such as Enterobacter cloacae, E. coli, Klebsiella oxytoca, and Klebsiella pneumoniae. Intriguingly, all of these microbes were also noted earlier among the emerging bacteria during this 11-year study period (Fig. 2; Fig. S1), and none of them were uniquely restricted to any of the reported oral diseases (Fig. S2).

## DISCUSSION

A central clinical question in oral infections is the identification of microbes associated with these infections. While previous studies have often reported mixed oral microbiota and presented a limited overview at the population level, this study focuses on oral bacteria at the species level and the epidemiology over an 11-year period of analysis. Here, we report the microbial composition of oral bacterial infections, as determined by MALDI-TOF MS technology, for the first time and confirm that dentoalveolar abscesses remained the most prevalent clinical diagnosis.

According to a recent nationwide survey in the United States, the number of periapical abscess-related emergency visits increased from 460,260 in 2008 to 545,693 in 2014, whereas the average hospital stay increased in duration from 2.96 days in 2008 to 4.5 days in 2014 (21). A similar observation has also been seen in our study, with dentoalveolar abscesses remained the main reason for microbiological testing over the study period. Our findings also revealed that microbes associated with dentoalveolar abscesses are diverse in nature, with a clear predominance of two genera, namely, Streptococcus spp. (in particular, *S. anginosus*) and *Prevotella* spp., which is consistent with the results of previous culture-dependent studies (22, 23). Moreover, *S. anginosus* alone, as a single species, accounted for over 20% of the bacterial infections that were reported in this study. The unique characteristic of the *S. anginosus* group (group D streptococci) that sets them apart from other pathogenic streptococci, such as S. pyogenes (group A streptococci) and Streptococcus agalactiae (group B streptococci), is their ability to cause abscesses in different body organs (24). However, some of the Gram-negative bacteria, such as *Veillonella* spp. that had been previously isolated from dentoalveolar abscesses via the traditional culturing method (22, 23) were not reported by MALDI-TOF MS in the current study. The absence of such bacteria could be attributed to culture-related factors, as a minimum concentration of microbes or a certain culture medium might be required for their clinical isolation and recognition.

Clinical microbiological examination investigates disease-causative and/or disease-associated agents and is mainly requested for difficult-to-treat cases. We present an epidemiological and longitudinal overview at the population level that has been lacking in the literature, in part because cultivated oral microbiology samples could not be classified to the species level before the MALDI-TOF MS analysis era. Monitoring the changes over time via trend analysis has given some insights into the context of the oral-systemic disease link. Given that severe tissue infections are seldom confined only to the locally affected area, it is sensible to monitor disease-associated microbes, as they may cross tissue barriers, evade immune surveillance, and thus disseminate to distant organs through the circulatory and lymphatic systems. If managed incorrectly, systemic infections and fatal conditions affecting the neural, cardiovascular, or pulmonary regions could occur (25, 26). Hence, microorganisms identified in oral infections have gained attention as potential biomarkers for some systemic disorders (18, 25).

Our findings demonstrate that a number of microorganisms have appeared much more frequently in oral infections over the past 11 years, specifically *S. anginosus*, S. mitis, S. sanguinis, S. epidermidis, E. corrodens, *A. aphrophilus*, E. faecalis, Klebsiella spp., *Granulicatella* spp., *Lactobacillus* spp., *Neisseria* spp., *Capnocytophaga* spp., and *Actinomyces* spp. Intriguingly, these microorganisms are frequently mentioned in the context of cancer development and progression, as well as in various systemic diseases, such as pericarditis and endocarditis (17, 18, 27, 28). Many of the gamma-proteobacteria reported here are also detected inside tumor lesions including in early precursors to invasive pancreatic cancer via microbiome and proteome analysis, which may interfere with cancer chemotherapy (29–31). *S. anginosus* and S. mitis remain common causes of abscesses in the liver and spleen, where immunosuppression, diabetes, and heavy alcohol use are among the known risk factors (24). The immunomodulatory effects of, for example, bacterial endotoxins, metabolites, and inflammatory mediators may modify the course of systemic health in (including but not limited to) cardiovascular diseases and malignancies along the gastrointestinal tract.

A recent report found that approximately 13% of the global cancer burden is attributed to microbial infections (32, 33), based on all known “oncomicrobes” to date. New evidence, however, suggests that the oncomicrobe category may be broader than previously thought and that “complicit” microbes specialised with microbial features such as genotoxin-mediated mutagenesis, may promote carcinogenesis (34). Deeper investigations to uncover such risk factors for earlier detection and intervention could be important in management of patients at risk.

Our trend analysis revealed an increase of the Gram-positive cocci pathogens in oral infections over the past 11 years. This supports recent findings from urinary tract and blood-based clinical microbiology and from epidemiological studies that report significant increases in Gram-positive infections over the past decade (35, 36), including multiple drug-resistant strains of Gram-positive cocci (37), which remain as a major global concern. This new information has substantial therapeutic implications, especially for odontogenic infections, including the possible use of NSAID/cyclooxygenase inhibitors to block the Gram-positive bacteria-associated NF-B overactivation in order to dampen tissue destruction (38). While Sweden remains in a favorable position compared to many other countries in terms of antibiotic resistance, the number of cases with a multiresistant Gram-positive infection is on the rise (39). The oral cavity can be a reservoir of such microbes, and oral health awareness could thus enable early intervention.

As estimated by Siqueira Jr. et al. via 16S rRNA sequencing (40), the microbial community of acute dental abscesses is a mix of 20 to 30 taxa per sample. However, data from the Human Oral Microbiome Database demonstrates that 24 to 46% of the oral taxa were uncultivated phylotypes. Thus, the clinical MALDI-TOF approach is a complementary alternative to 16S rRNA sequencing, as up to seven unique viable species could be classified simultaneously in a clinical routine laboratory. Possibilities for experimental functional studies of live oral microbiota and the whole-genomic sequencing of various specialized ecologies could improve the current understanding of human oral microbiota. The downside of this clinical approach is that some species are difficult to cultivate and require special culture conditions. Hence, our frequently reported live core species did not exactly meet the taxa reported earlier, (i.e., F. nucleatum, *Porphyromonas* spp., Treponema spp., Parvimonas micra, *Tannerela forsythia*, and *Dialister* spp.) (40).

Jaw osteonecrosis is another oral condition, and it is caused by radiation-induced injury or medication-induced osteonecrosis (41). In our study, samples from patients diagnosed with jaw osteonecrosis were found to contain a high predominance of facultative anaerobes with a high frequency of Streptococcus spp. and *Prevotella* spp. These findings support the findings of reports from recent research (42, 43). Moreover, microbes that are thought to be the leading causes of delayed wound healing in osteonecrosis (44), such as Streptococcus spp., S. aureus, and Pseudomonas aeruginosa, have also been found in our study. Contrary to other studies (42, 43), our findings showed lower levels of *Actinomyces* spp. and P. aeruginosa in osteonecrosis samples, as well as a lack of *Veillonella* species. The absence of *Veillonella* spp., as well as the reporting of *Actinomyces* spp. and P. aeruginosa at a lower frequency, could be attributed to differences in the site of the harvested samples, history of antibiotic therapy, overgrowth of concomitant microorganisms, or culturing methods.

Another type of oral infection is jaw osteomyelitis, which represents an inflammation of the jawbone, triggered by microbial infection (45). In this study, we found Streptococcus, Staphylococcus, *Prevotella*, *Enterococcus*, and *Cutibacterium* species, with a greater proportion of facultative anaerobes being found in the jaw osteomyelitis samples. These findings are in line with those of earlier reports (45, 46). However, while Parvimonas micra in jaw osteomyelitis has previously been described (45), our findings did not confirm the presence of this isolate. This could be explained by the challenges associated with the cultivation of this microorganism.

There is currently limited data on the microbiology of odontogenic maxillary sinusitis. Earlier studies showed that most microorganism populations consist of mixed aerobic and anaerobic species (10). In line with this, our findings revealed that samples from odontogenic maxillary sinusitis may consist of a range of different bacterial genera of aerobic (Streptococcus spp., Staphylococcus spp., and *Neisseria* spp.) and anaerobic (*Prevotella* spp. and Haemophilus spp.) bacteria. A relatively similar microbial composition to that found in dentoalveolar abscesses is also noted here. For the periapical pathosis samples, Streptococcus spp. and E. faecalis appear to be the most frequent. The latter is commonly associated with failed endodontic therapy and postendodontic treatment complications (47). Meanwhile, E. faecalis has a high antimicrobial resistance, can survive in harsh environments with a limited nutrient supply, and induces hydroxyapatite deposition and biofilm calcification as resistance mechanisms (47). Other frequently reported microbes found here include E. coli, *Prevotella* spp., *Actinomyces* spp., and *Cutibacterium* spp., all of which are in line with earlier research (48).

The current study’s microbiological findings from the periodontal disease samples are consistent with those of previous reports (49), in which *Prevotella* spp., *Capnocytophaga* spp., and *Eikenella* spp. are frequently reported. According to Haffajee and Socransky’s classification (50), these microbes are regarded as strong pathogens in periodontal diseases, indicating plaque-induced gingivitis or peri-implantitis rather than chronic periodontitis conditions. However, the absence of *Fusobacteria* spp. in the periodontal samples could be explained by the relatively small sample size of this group and the category of the periodontal infections submitted to this analysis (i.e., acute rather than chronic cases). Other factors which may have affected our results include the competitive metabolic microenvironments, cross-feeding, fastidious microorganisms, or overgrowth of single microorganisms during the cultivation process (51). We opt to present clinical oral microbiology from a large, real-world, clinical cohort, as we believe it could bring insights into the context of the clinical management of oral infections (that is, the selection of antimicrobial therapy, the determination of whether hospitalization is necessary, and the change of mortality and progression of infections).

One of the main strengths of our study is that it is the first study to describe the microbial profile of different severe oral infections at the population level, using MALDI-TOF MS technology, with a relatively large sample size. This technology allows for the precise identification of microorganisms that remained unidentifiable by conventional phenotypic methods, is rapidly becoming a cost-effective alternative to 16S rRNA gene sequencing, and presents with unquestioned reliability (20). We also analyzed for the first-time longitudinal trends in oral infections over an 11-year study period, core microbes across different oral infections, and various sampling materials. Further, the adopted culturing technique and microbial identification procedures were conducted using a standardized laboratory protocol throughout the whole 11-year investigation period.

We acknowledge that this study has a number of limitations. First, it is retrospective in design, which limits the evaluation of factors that are not regularly documented, including histopathological evaluations and the use of antibiotics. Another limitation is that clinical details, including treatment outcomes, were not collected. Combining this information with microbiology findings will shed more light on the current study and help us to understand the potential emerging pathogenicity of oral microbes. Moreover, the microbial load/density and antimicrobial susceptibility results were not integrated into the current microbiological database, and the microbial findings in the current study are based exclusively on reports of bacterial outputs. Since oral infections are typically polymicrobial in nature, combining the MALDI-TOF results with a 16S rRNA gene sequencing analysis in future studies will provide complementary insights into the microbial ecology of these infections. Lastly, as this study was conducted in the capital of Sweden, our results may not be generalizable to other parts of the world. Comparisons of microbial trends with other geographical areas thus remain to be performed.

## MATERIALS AND METHODS

### Ethical approval.

The study was approved by the regional ethical review board in Stockholm, Sweden (Dnr 2021-03974) and was deemed to be in compliance with good clinical practice, the principles of the Declaration of Helsinki, and the standards of Karolinska University Hospital.

### Sample identification.

The study was carried out retrospectively by using the sample data/records from within the Qlik-View software system (Qlik, Lund, Sweden) at Karolinska University Hospital’s Clinical Microbiology lab, spanning the years from 2010 to 2020. All clinical samples with microbial growth were subjected to a MALDI-TOF MS (Bruker, Daltonik, GmbH, Bremen, Germany) analysis. The exported data were sorted in Excel and filtered with the following key terms: dental, tooth, oral care, gum, mouth, jaw, craniofacial, and maxillofacial. All oral infection samples submitted by dental clinics and hospitals in Stockholm County were collected. In addition to the microbiological data, clinical variables, including the clinical diagnosis (dentoalveolar abscesses, jaw osteomyelitis, jaw osteonecrosis, odontogenic maxillary sinusitis, periradicular pathosis, and periodontal diseases), involved facial space/s, and sampling materials (abscess, mucosa swabs, or tissues) were collected, as were general variables, such as the age and gender of each patient.

### Culturing technique.

All samples were cultured on blood, chocolate, cystine lactose electrolyte deficient (CLED), and Brucella laked blood agar plates and were incubated under aerobic and anaerobic conditions. For the aerobic conditions, the blood and CLED plates were incubated at 37°C in a relevant atmosphere, while the chocolate plate was incubated at 37°C in a 5 to 10% CO_2_ environment. For the anaerobic conditions, an anaerobic chamber (Anaerocult A; Merck, Darmstadt, Germany) was used to incubate the blood agar and Brucella lacked blood agar plate with kanamycin and vancomycin at 37°C. All of the agar plates were read twice, after 24 h and 48 h for the aerobic plates, and after 48 h and 6 days for the anaerobic plates. After microbial isolation, the colonies were conveyed to MALDI-TOF MS identification.

### MALDI-TOF MS identification.

A well-formed colony from the culture plates was directly spread on the MALDI targeted plate. After adding formic acid (1 μL of a 70% solution) and air-drying, 1 μL of matrix solution was applied at room temperature. Using the MALDI Biotyper (version 3.0) and FlexControl (version 3.3) software packages, the ionization and acceleration of the agent macromolecules embedded in small matrix molecules in an electromagnetic field of 10 to 30 k permitted the identification of different flight times. The microbial spectra were then matched against the references in the database. The obtained score value (log) data ranged from ≥2 for species-level identification and ≥1.7 for genus-level identification. Microbes with a score of <1.7 were described by their morphology and were classified as others in this study.

### Statistical analysis.

The data were analyzed as primary data. To minimize potential technical errors and to increase the ecological relevance of oral infections, data filtration processes were applied by removing bacteria with a count of less than 2. The descriptive analysis, average number of identified isolates, male or female origin, and age groups were reported as means (± standard deviations) and proportions (%). Microbial frequencies were reported as proportions of phyla, genera, species, gram staining, morphology, oxygen metabolisms, different clinical diagnoses, and sampling materials. For the inferential analyses, a multivariable logistic regression analysis, modeling the odds of monomicrobial or polymicrobial culture, was conducted to identify independent predictors of the clinical diagnosis, age group, gender, and sample material. First, the multivariable analysis was used to calculate partially adjusted odds ratios (OR) and 95% confidence intervals (CI), which were adjusted for the age group, gender, and sample material. The final adjusted analysis took into consideration the simultaneous effects of relevant predictors to investigate their independent impacts on monomicrobial and polymicrobial cultures. Additionally, a linear regression model was used to examine trends of microbial frequency from 2010 to 2020. Based on the R values, the trend was described as increased, reduced, or steady. A hierarchical clustering heat map was created, using the R package pheatmap (R-4.0.4; Core Team, 2021, Vienna, Austria), using the Euclidean distance measure and the Ward clustering algorithm. The diversity of the detected microorganisms was plotted using Venn diagrams. The data were analyzed using SPSS version 24.0 (IBM Corp., Armonk, NY, USA). A *P* value of <0.05 was considered to be indicative of a statistically significant result.

### Data availability.

Data will be made available upon request to the corresponding author, following the publication of the present article.

## References

[1] Peres MA, Macpherson LMD, Weyant RJ, Daly B, Venturelli R, Mathur MR, Listl S, Celeste RK, Guarnizo-Herreño CC, Kearns C, Benzian H, Allison P, Watt RG. 2019. Oral diseases: a global public health challenge. Lancet 394:249–260. doi:10.1016/S0140-6736(19)31146-8. 31327369

[2] 2020. Global burden of 369 diseases and injuries in 204 countries and territories, 1990–2019: a systematic analysis for the Global Burden of Disease Study 2019. Lancet 396:1204–1222. doi:10.1016/S0140-6736(20)30925-9. 33069326 PMC7567026

[3] Dahlén G. 2009. Non-odontogenic infections in dentistry. Periodontol 2000 49:7–12. doi:10.1111/j.1600-0757.2008.00298.x. 19152523 PMC7167770

[4] Ogle OE. 2017. Odontogenic infections. Dent Clin North Am 61:235–252. doi:10.1016/j.cden.2016.11.004. 28317564

[5] Manoil D, Al-Manei K, Belibasakis GN. 2020. A systematic review of the root canal microbiota associated with apical periodontitis: lessons from next-generation sequencing. Proteomics Clin Appl 14:e1900060. doi:10.1002/prca.201900060. 31950679

[6] Gomes B, Herrera DR. 2018. Etiologic role of root canal infection in apical periodontitis and its relationship with clinical symptomatology. Braz Oral Res 32:e69. 30365610 10.1590/1807-3107bor-2018.vol32.0069

[7] Levi ME, Eusterman VD. 2011. Oral infections and antibiotic therapy. Otolaryngol Clin North Am 44:57–78, v. doi:10.1016/j.otc.2010.10.003. 21093623

[8] Lypka M, Hammoudeh J. 2011. Dentoalveolar infections. Oral Maxillofac Surg Clin North Am 23:415–424. doi:10.1016/j.coms.2011.04.010. 21602052

[9] Moratin J, Freudlsperger C, Metzger K, Braß C, Berger M, Engel M, Hoffmann J, Ristow O. 2021. Development of osteomyelitis following dental abscesses-influence of therapy and comorbidities. Clin Oral Invest 25:1395–1401. doi:10.1007/s00784-020-03447-6. 32638128

[10] Psillas G, Papaioannou D, Petsali S, Dimas GG, Constantinidis J. 2021. Odontogenic maxillary sinusitis: a comprehensive review. J Dent Sci 16:474–481. doi:10.1016/j.jds.2020.08.001. 33384837 PMC7770314

[11] Yue Yi EK, Siew Ying AL, Mohan M, Menon RK. 2021. Prevalence of postoperative infection after tooth extraction: a retrospective study. Int J Dent 2021:6664311. doi:10.1155/2021/6664311. 34211554 PMC8208874

[12] He L, Sun X, Liu Z, Qiu Y, Niu Y. 2020. Pathogenesis and multidisciplinary management of medication-related osteonecrosis of the jaw. Int J Oral Sci 12:30. doi:10.1038/s41368-020-00093-2. 33087699 PMC7578793

[13] Kawashita Y, Funahara M, Yoshimatsu M, Nakao N, Soutome S, Saito T, Umeda M. 2018. A retrospective study of factors associated with the development of oral candidiasis in patients receiving radiotherapy for head and neck cancer: is topical steroid therapy a risk factor for oral candidiasis? Medicine (Baltimore, MD) 97:e13073. doi:10.1097/MD.0000000000013073. PMC6221665 30383690

[14] Nishii M, Soutome S, Kawakita A, Yutori H, Iwata E, Akashi M, Hasegawa T, Kojima Y, Funahara M, Umeda M, Komori T. 2020. Factors associated with severe oral mucositis and candidiasis in patients undergoing radiotherapy for oral and oropharyngeal carcinomas: a retrospective multicenter study of 326 patients. Support Care Cancer 28:1069–1075. doi:10.1007/s00520-019-04885-z. 31177394

[15] Shuster A, Reiser V, Trejo L, Ianculovici C, Kleinman S, Kaplan I. 2019. Comparison of the histopathological characteristics of osteomyelitis, medication-related osteonecrosis of the jaw, and osteoradionecrosis. Int J Oral Maxillofac Surg 48:17–22. doi:10.1016/j.ijom.2018.07.002. 30054185

[16] Willis JR, Gabaldón T. 2020. The human oral microbiome in health and disease: from sequences to ecosystems. Microorganisms 8:308. doi:10.3390/microorganisms8020308. 32102216 PMC7074908

[17] Irfan M, Delgado RZR, Frias-Lopez J. 2020. The oral microbiome and cancer. Front Immunol 11:591088. doi:10.3389/fimmu.2020.591088. 33193429 PMC7645040

[18] Peng X, Cheng L, You Y, Tang C, Ren B, Li Y, Xu X, Zhou X. 2022. Oral microbiota in human systematic diseases. Int J Oral Sci 14:14. doi:10.1038/s41368-022-00163-7. 35236828 PMC8891310

[19] Dominguez-Bello MG, Godoy-Vitorino F, Knight R, Blaser MJ. 2019. Role of the microbiome in human development. Gut 68:1108–1114. doi:10.1136/gutjnl-2018-317503. 30670574 PMC6580755

[20] Singhal N, Kumar M, Kanaujia PK, Virdi JS. 2015. MALDI-TOF mass spectrometry: an emerging technology for microbial identification and diagnosis. Front Microbiol 6:791. doi:10.3389/fmicb.2015.00791. 26300860 PMC4525378

[21] Rampa S, Veeratrishul A, Raimondo M, Connolly C, Allareddy V, Nalliah RP. 2019. Hospital-based emergency department visits with periapical abscess: updated estimates from 7 years. J Endod 45:250–256. doi:10.1016/j.joen.2018.12.004. 30803531

[22] Siqueira JF, Jr, Rôças IN. 2013. Microbiology and treatment of acute apical abscesses. Clin Microbiol Rev 26:255–273. doi:10.1128/CMR.00082-12. 23554416 PMC3623375

[23] Böttger S, Zechel-Gran S, Schmermund D, Streckbein P, Wilbrand J-F, Knitschke M, Pons-Kühnemann J, Hain T, Weigel M, Howaldt H-P, Domann E, Attia S. 2021. Microbiome of odontogenic abscesses. Microorganisms 9:1307. doi:10.3390/microorganisms9061307. 34208451 PMC8234849

[24] Issa E, Salloum T, Tokajian S. 2020. From normal flora to brain abscesses: a review of Streptococcus intermedius. Front Microbiol 11:826. doi:10.3389/fmicb.2020.00826. 32457718 PMC7221147

[25] Zhang Y, Wang X, Li H, Ni C, Du Z, Yan F. 2018. Human oral microbiota and its modulation for oral health. Biomed Pharmacother 99:883–893. doi:10.1016/j.biopha.2018.01.146. 29710488

[26] Mannan S, Tordik PA, Martinho FC, Chivian N, Hirschberg CS. 2021. Dental abscess to septic shock: a case report and literature review. J Endod 47:663–670. doi:10.1016/j.joen.2020.12.016. 33422573

[27] Vogkou CT, Vlachogiannis NI, Palaiodimos L, Kousoulis AA. 2016. The causative agents in infective endocarditis: a systematic review comprising 33,214 cases. Eur J Clin Microbiol Infect Dis 35:1227–1245. doi:10.1007/s10096-016-2660-6. 27170145

[28] Karpiński TM. 2019. Role of oral microbiota in cancer development. Microorganisms 7:20. doi:10.3390/microorganisms7010020. 30642137 PMC6352272

[29] Gaiser RA, Halimi A, Alkharaan H, Lu L, Davanian H, Healy K, Hugerth LW, Ateeb Z, Valente R, Fernández Moro C, Del Chiaro M, Sällberg Chen M. 2019. Enrichment of oral microbiota in early cystic precursors to invasive pancreatic cancer. Gut 68:2186–2194. doi:10.1136/gutjnl-2018-317458. 30872392 PMC6872446

[30] Geller LT, Barzily-Rokni M, Danino T, Jonas OH, Shental N, Nejman D, Gavert N, Zwang Y, Cooper ZA, Shee K, Thaiss CA, Reuben A, Livny J, Avraham R, Frederick DT, Ligorio M, Chatman K, Johnston SE, Mosher CM, Brandis A, Fuks G, Gurbatri C, Gopalakrishnan V, Kim M, Hurd MW, Katz M, Fleming J, Maitra A, Smith DA, Skalak M, Bu J, Michaud M, Trauger SA, Barshack I, Golan T, Sandbank J, Flaherty KT, Mandinova A, Garrett WS, Thayer SP, Ferrone CR, Huttenhower C, Bhatia SN, Gevers D, Wargo JA, Golub TR, Straussman R. 2017. Potential role of intratumor bacteria in mediating tumor resistance to the chemotherapeutic drug gemcitabine. Science 357:1156–1160. doi:10.1126/science.aah5043. 28912244 PMC5727343

[31] Halimi A, Gabarrini G, Sobkowiak MJ, Ateeb Z, Davanian H, Gaiser RA, Arnelo U, Valente R, Wong AYW, Moro CF, Del Chiaro M, Özenci V, Chen MS. 2021. Isolation of pancreatic microbiota from cystic precursors of pancreatic cancer with intracellular growth and DNA damaging properties. Gut Microbes 13:1983101. doi:10.1080/19490976.2021.1983101. 34816784 PMC8632270

[32] Wade WG. 2013. The oral microbiome in health and disease. Pharmacol Res 69:137–143. doi:10.1016/j.phrs.2012.11.006. 23201354

[33] Zarco MF, Vess TJ, Ginsburg GS. 2012. The oral microbiome in health and disease and the potential impact on personalized dental medicine. Oral Dis 18:109–120. doi:10.1111/j.1601-0825.2011.01851.x. 21902769

[34] Sepich-Poore GD, Zitvogel L, Straussman R, Hasty J, Wargo JA, Knight R. 2021. The microbiome and human cancer. Science 371. doi:10.1126/science.abc4552. PMC8767999 33766858

[35] Gajdács M, Ábrók M, Lázár A, Burián K. 2020. Increasing relevance of Gram-positive cocci in urinary tract infections: a 10-year analysis of their prevalence and resistance trends. Sci Rep 10:17658. doi:10.1038/s41598-020-74834-y. 33077890 PMC7573585

[36] Zhu Q, Yue Y, Zhu L, Cui J, Zhu M, Chen L, Yang Z, Liang Z. 2018. Epidemiology and microbiology of Gram-positive bloodstream infections in a tertiary-care hospital in Beijing, China: a 6-year retrospective study. Antimicrob Resist Infect Control 7:107. doi:10.1186/s13756-018-0398-x. 30202520 PMC6122739

[37] Huang L, Zhang R, Hu Y, Zhou H, Cao J, Lv H, Chen S, Ding S, Chen G. 2019. Epidemiology and risk factors of methicillin-resistant Staphylococcus aureus and vancomycin-resistant enterococci infections in Zhejiang China from 2015 to 2017. Antimicrob Resist Infect Control 8:90. doi:10.1186/s13756-019-0539-x. 31164979 PMC6543620

[38] Tominari T, Sanada A, Ichimaru R, Matsumoto C, Hirata M, Itoh Y, Numabe Y, Miyaura C, Inada M. 2021. Gram-positive bacteria cell wall-derived lipoteichoic acid induces inflammatory alveolar bone loss through prostaglandin E production in osteoblasts. Sci Rep 11:13353. doi:10.1038/s41598-021-92744-5. 34172796 PMC8233430

[39] Public Health Agency of Sweden. Availability of antibiotics. https://wwwfolkhalsomyndighetense/the-public-health-agency-of-sweden/communicable-disease-control/antibiotics-and-antimicrobial-resistance/availability-of-antibiotics/2022.

[40] Siqueira JF, Rôças IN, Jr. 2022. Rôças IN: present status and future directions: microbiology of endodontic infections. Int Endod J 55 Suppl 3:512–530. doi:10.1111/iej.13677. 34958494

[41] American College of Rheumatology. 2022. Osteonecrosis of the jaw (ONJ). https://wwwrheumatologyorg/I-Am-A/Patient-Caregiver/Diseases-Conditions/Osteonecrosis-of-the-Jaw-ONJ .

[42] Panya S, Fliefel R, Probst F, Tröltzsch M, Ehrenfeld M, Schubert S, Otto S. 2017. Role of microbiological culture and polymerase chain reaction (PCR) of actinomyces in medication-related osteonecrosis of the jaw (MRONJ). J Craniomaxillofac Surg 45:357–363. doi:10.1016/j.jcms.2017.01.006. 28162845

[43] Zirk M, Wenzel C, Buller J, Zöller JE, Zinser M, Peters F. 2019. Microbial diversity in infections of patients with medication-related osteonecrosis of the jaw. Clin Oral Invest 23:2143–2151. doi:10.1007/s00784-018-2655-z. 30276516

[44] Wei X, Pushalkar S, Estilo C, Wong C, Farooki A, Fornier M, Bohle G, Huryn J, Li Y, Doty S, Saxena D. 2012. Molecular profiling of oral microbiota in jawbone samples of bisphosphonate-related osteonecrosis of the jaw. Oral Dis 18:602–612. doi:10.1111/j.1601-0825.2012.01916.x. 22443347 PMC7167636

[45] Dym H, Zeidan J. 2017. Microbiology of acute and chronic osteomyelitis and antibiotic treatment. Dent Clin North Am 61:271–282. doi:10.1016/j.cden.2016.12.001. 28317566

[46] Lucidarme Q, Lebrun D, Vernet-Garnier V, Le Gall J, Diallo S, Mauprivez C, Derruau S. 2022. Chronic osteomyelitis of the jaw: pivotal role of microbiological investigation and multidisciplinary management-a case report. Antibiotics (Basel) 11:568. doi:10.3390/antibiotics11050568. 35625212 PMC9137754

[47] Alghamdi F, Shakir M. 2020. The Influence of Enterococcus faecalis as a dental root canal pathogen on endodontic treatment: a systematic review. Cureus 12:e7257. doi:10.7759/cureus.7257. 32292671 PMC7152576

[48] Bronzato JD, Bomfim RA, Hayasida GZP, Cúri M, Estrela C, Paster BJ, Gomes B. 2021. Analysis of microorganisms in periapical lesions: a systematic review and meta-analysis. Arch Oral Biol 124:105055. doi:10.1016/j.archoralbio.2021.105055. 33588190

[49] Harvey JD. 2017. Periodontal microbiology. Dent Clin North Am 61:253–269. doi:10.1016/j.cden.2016.11.005. 28317565

[50] Haffajee AD, Socransky SS. 2005. Microbiology of periodontal diseases: introduction. Periodontol 2000 38:9–12. doi:10.1111/j.1600-0757.2005.00112.x. 15853934

[51] Wolcott R, Costerton JW, Raoult D, Cutler SJ. 2013. The polymicrobial nature of biofilm infection. Clin Microbiol Infect 19:107–112. doi:10.1111/j.1469-0691.2012.04001.x. 22925473

