# Supplementary material for: Clinical Microbial Identification of Severe Oral Infections by MALDI-TOF Mass Spectrometry in Stockholm County: an 11-Year (2010 to 2020) Epidemiological Investigation
Source: Microbiol Spectr. 2022 Nov 24;10(6):e02487-22. doi: 10.1128/spectrum.02487-22 (PMC9769555; doi:10.1128/spectrum.02487-22)
Supplement: Supplemental file 1 — Supplemental material. Download spectrum.02487-22-s0001.pdf, PDF file, 3.6 MB [file spectrum.02487-22-s0001.pdf]

Suppl. Figure 1

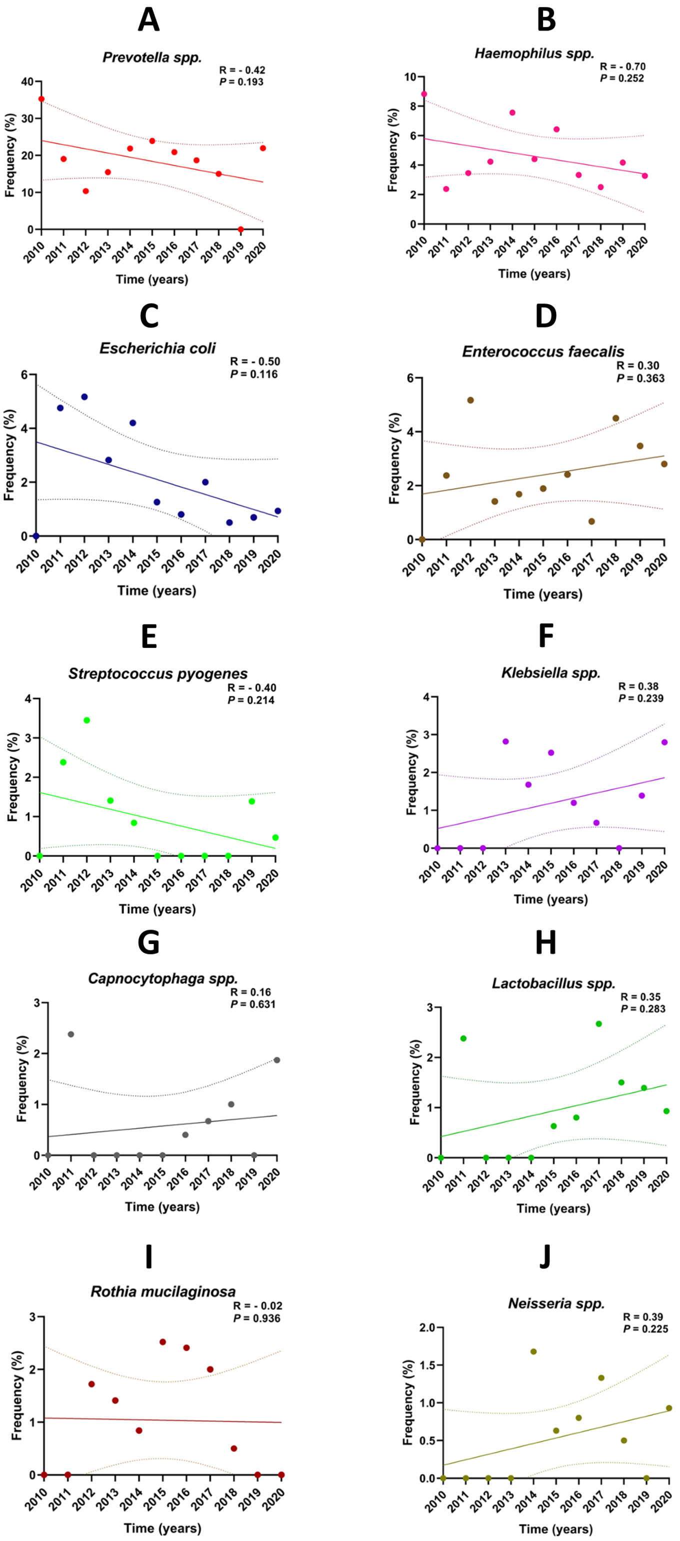

Supplementary Figure 1: Linear regression analysis on microbial trends over the 11-year study period of (A) *Prevotella* spp., (B) *Haemophilus* spp., (C) *Escherichia coli,* (D) *Enterococcus faecalis,* (E) *Streptococcus pyogenes,* (F) *Klebsiella* spp., (G) *Capnocytophaga* spp., (H) *Lactobacillus* spp., (I) *Rothia mucilaginosa,* and (J) *Neisseria* spp., *P*-values >0.05 are considered as not significant.

Suppl. Figure 2

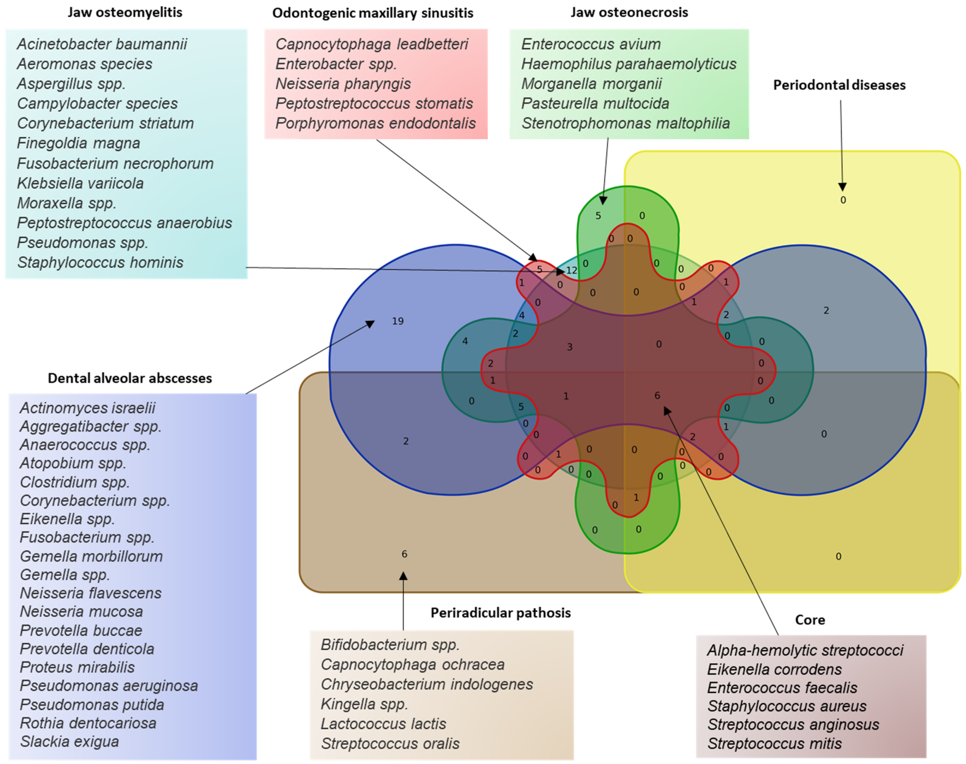

Supplementary Figure 2: Venn diagram depicts the unique- respectively core microbes found in oral infection samples that are classified by their clinical diagnosis.
